# A systematic review and meta-analysis of the effects of clinical pathways on length of stay, hospital costs and patient outcomes

**DOI:** 10.1186/1472-6963-8-265

**Published:** 2008-12-19

**Authors:** Thomas Rotter, Joachim Kugler, Rainer Koch, Holger Gothe, Sabine Twork, Jeroen M van Oostrum, Ewout W Steyerberg

**Affiliations:** 1 Department of Public Health, Dresden Medical School, University of Dresden, Dresden, Germany; 2 Institute of Medical Statistics, University of Dresden, Dresden, Germany; 3 IGES Institute GmbH, Berlin, Germany; 4 Econometric Institute, Erasmus University Rotterdam, Rotterdam, The Netherlands; 5 Center for Medical Decision Making, Department of Public Health, Erasmus MC University Medical Center Rotterdam, Rotterdam, The Netherlands

## Abstract

**Background:**

To perform a systematic review about the effect of using clinical pathways on length of stay (LOS), hospital costs and patient outcomes. To provide a framework for local healthcare organisations considering the effectiveness of clinical pathways as a patient management strategy.

**Methods:**

As participants, we considered hospitalized children and adults of every age and indication whose treatment involved the management strategy "clinical pathways". We include only randomised controlled trials (RCT) and controlled clinical trials (CCT), not restricted by language or country of publication. Single measures of continuous and dichotomous study outcomes were extracted from each study. Separate analyses were done in order to compare effects of clinical pathways on length of stay (LOS), hospital costs and patient outcomes. A random effects meta-analysis was performed with untransformed and log transformed outcomes.

**Results:**

In total 17 trials met inclusion criteria, representing 4,070 patients. The quality of the included studies was moderate and studies reporting economic data can be described by a very limited scope of evaluation. In general, the majority of studies reporting economic data (LOS and hospital costs) showed a positive impact. Out of 16 reporting effects on LOS, 12 found significant shortening. Furthermore, in a subgroup-analysis, clinical pathways for invasive procedures showed a stronger LOS reduction (weighted mean difference (WMD) -2.5 days versus -0.8 days)).

There was no evidence of differences in readmission to hospitals or in-hospital complications. The overall Odds Ratio (OR) for re-admission was 1.1 (95% CI: 0.57 to 2.08) and for in-hospital complications, the overall OR was 0.7 (95% CI: 0.49 to 1.0). Six studies examined costs, and four showed significantly lower costs for the pathway group. However, heterogeneity between studies reporting on LOS and cost effects was substantial.

**Conclusion:**

As a result of the relatively small number of studies meeting inclusion criteria, this evidence base is not conclusive enough to provide a replicable framework for all pathway strategies. Considering the clinical areas for implementation, clinical pathways seem to be effective especially for invasive care. When implementing clinical pathways, the decision makers need to consider the benefits and costs under different circumstances (e.g. market forces).

## Background

Clinical pathways represent a form of "cookbook medicine" that many perceive as an appropriate tool that contributes to quality management, cost-cutting and patient satisfaction.

For the aim of this review, clinical pathways are defined as complex interventions consisting of a number of components based on the best available evidence and guidelines for specific conditions [1]. A clinical pathway defines the sequencing and timing of health interventions and should be developed through the collaborative effort of physicians, nurses, pharmacists, and other associated health professionals [2]. Clinical pathways aim to minimize delays and maximize resource utilization and quality of care [1]. They are also referred to as "integrated care pathways", "critical pathways", "care plans", "care paths", "care maps" and "care protocols".

The effectiveness of clinical pathways is under debate. However, especially in the US, up to 80 percent of hospitals already use clinical pathways for at least some indications [3]. A number of primary studies considered the effectiveness of clinical pathways, but results are inconsistent and suffer from various biases [4-7]. Only one systematic review has been performed, specifically for stroke patients [8]. Narrative reviews are more common, which often rely on "expert opinions" [9-11].

We perform a systematic review and a random effects meta-analysis to assess whether clinical pathways improved the outcome measures "length of stay (LOS)", "hospital costs" and "quality of care" when compared to standard care. By performing a systematic review and meta-analysis we are able to present the available evidence in a substantiated and concise way, in order to provide a framework for local healthcare organisations considering the effectiveness of clinical pathways.

## Methods

We followed the methods of the Cochrane Collaboration [12] with some modifications, mainly concerning presentation of meta-analytic results.

### Study selection criteria

As potential patient samples we considered hospitalized children and adults of every age and indication, whose treatment involved the management strategy "clinical pathways". Given the problem that there are variations in the terminology used in the current research [13], we defined minimum "inclusion criteria" for meeting our clinical pathway definition (see Table 1). Based on our definition (see background), we developed a pre-specified, three operational pathway criteria as follows: 1) multidisciplinary (two or multiple clinical professions involved), 2) protocol or algorithm based (i.e. structured care plan/treatment-protocol or algorithm) and finally, 3) evidence based (pathway components were minimally based on one RCT or best practice guidelines). Every pathway characteristic could be met as (1) "yes" criterion; (2) "not sure" because of poor reporting and the failure to contact the principal author or (3) "criterion not met." If one or more pathway criteria selected is not met, then we excluded the study.

**Table 1:** Pathway characteristics and quality outcome measures of studies included

**Pathway**	**Charac-teristics**			**Quality Measure**	**Pathway [n/N]**	**Control [n/N]**
**Study-ID**	**multi-disciplinary**	**evidence-based**	**protocol/algorithm based**		**Counts and rates are presented in natural units and as percentages as far as reported**	**N = numer of participants n = number of events (%) = percentage**

**Invasive Care**						

Grines, CL	X	X	X	In-hospital complications	20/237 (8.4%)	20/234 (8.5%) N.S.
1998				Re-hospitalisation (6 months)	10/237 (4.2%)	9/234 (3.9%) N.S.
Swanson, CE	X	Not	X	Hospital mortality	2/38 (5.2%)	2/33 (6.1%) N.S.
1998		sure		Mean Modified Barthel Index	92.8	85.6 (p < 0.05)
Dowsey, MM	X	X	X	In-hospital complications	10/92 (10.8%)	20/71 (28.1%) (p < 0.05)
1999				Re-hospitalisation (3 months)	1/92 (1.1%)	0/71 (0%) N.S.
Choong, PF	X	X	X	In-hospital complications	10/55 (18.2%)	14/56 (25.0%) N.S.
2000				Re-hospitalisation (28 days)	2/55 (3.6%)	6/56 (10.7%) N.S.
Aizawa, T	X	X	X	In-hospital complications	1/32 (3.1%)	2/37 (5.4%) N.S.
2002				Re-hospitalisation (6 months)	1/32 (3.1%)	0/37 (0%) N.S.
Kiyama, T	X	X	X	In-hospital complications	3/47 (6.4%)	5/38 (13.2%) N.S.
2003						
Hirao, M	X	X	X	In-hospital complications	19/53 (35.8%)	17/50 (34.0%) N.S.
2005				Re-hospitalisation (6 months)	0/53 (0%)	0/50 (0%)
**Non-Invasive Care**						
Falconer, JA	X	Not	X	Mortality (12 months)	N.S.	N.S.
1993		Sure		Re-hospitalisation (12 months)	N.S.	N.S.
				Cognitive and functional scores (0–100)	N.S.	N.S.
				Patient satisfaction	7.7 (SD 2.6)	8.8 (SD 1.7) (p < 0.05)
Gomez, MA	X	X	X	Complete and graded exercise test	44/50 (88.0%)	15/50 (30.0%)
1996						
Roberts, RR	X	X	X	Hospitalised patients as %	(45.1%)	(100%)
1997				Re-hospitalisation (8 weeks)	5/82 (6.1%)	4/83 (4.8%) N.S.
Johnson, KB 2000	X	X	X	Unscheduled clinic visits; no hospital re-admission (2 weeks)	0/55 (0%)	2/55 (3.6%)
Kollef, HM	X	X	X	In-hospital complications	9/239 (3.8%)	13/250 (5.2%) N.S.
2000				Hospital mortality	5/239 (2.1%)	8/250 (3.2%) N.S.
Marrie, TJ 2000	X	X	X	Absolute difference in rates (ARR) between pathway and control		1-sided 95% CI upper limit:
				In-hospital complications	(0.6%)	(4.6%) N.S.
				Re-hospitalisation (6 weeks)	(0.7%)	(3.6%) N.S.
				Mortality (6 weeks)	(-0.1%)	(2.5%) N.S.
Sulch, D	X	X	X	Median Barthel Index Score (26 weeks)	17	17 N.S.
1999				Mortality (26 weeks)	10/76 (13.2%)	6/76 (7.9%) N.S.
Kim, MH	X	X	X	Complications until follow-up (27 days)	1/9 (11.1%)	1/9 (11.1%) N.S.
2002				Re-hospitalisation (27 days)	2/9 (22.2%)	0/9 (0%) N.S.
Chen, SH 2004	X	X	X	Emergency room usage (not comparable with in-hospital complications)	3/20 (15.0%)	13/22 (59.1%) (p < 0.05)
				Re-hospitalisation (3 months)	N.S.	N.S.
Usui, K 2004	X	X	X	Not reported		

Legend: Every pathway characteristic could be met as (1) "yes" criterion; (2) "not sure" because of poor reporting and the failure to contact the principal author or (3) "criterion not met." If one or more pathway criteria selected is/are not met, then we excluded the study. Due to poor reporting some quality measures are presented only as percentages or mean scores with or without associated standard deviations (SD). Some quality measures were only reported as statistical significant (i.e. p < 0.05) or not significant (N.S.) and any other data were missing.

Please note, additional information relating to the included studies that matched these requirements or differ from each other, are given in the results section of this review.

The setting definition covered the whole range of services offered by the clinical (out- and in-patient) as well as in the in-patient rehabilitation sector. We only gathered robust evidence and limited our study selection to randomised controlled trials (RCT) and controlled clinical trials (CCT) including methodological quality criteria (please see "quality assessment and data analyses").

We considered every objective economic and patient outcome for inclusion. We pre-defined (1) in-hospital complications as a secondary disease or adverse medical occurrence during hospitalization [14] and (2) we defined re-hospitalization as a readmission within a specified follow up period of an index admission.

### Data sources and search strategy

We performed specialised searches of the Medline database (1966–2006), Embase (1980–2006), Cinahl (1982–2006), Global Health (1973–2006), and the specialised Cochrane register (including NHS EED and HTA Database; last update: 13.11.06), not restricted by language or country. We used free text words (tw), medical subject headings (MeSH terms -/-) or exploded MeSH terms for our MEDLINE literature search. This controlled vocabulary was adapted (as much as possible) to the indexation (thesaurus) of all other databases included in this review. We demonstrate our "clinical pathway search strategy" with the MEDLINE inquiry (Table 2).

**Table 2:** Clinical pathway search strategy Ovid Medline: 1966 to November Week 2 2006

1.	Critical Pathways/
2.	(clinical path$ or critical path$ or care path$ or care map$).tw.
3.	exp Guidelines/
4.	Health Planning Guidelines/
5.	Guideline Adherence/
6.	(guideline? adj2 introduc$ or issu$ or impact or effect? or disseminat$ or distribut$)).tw.
7.	nursing protocol$.tw.
8.	(professional standard$ or professional protocol or professional care map).tw.
9.	(practice guidelin$ or practice protocol$ or clinical practice guideline$).tw.
10.	guideline.pt.
11.	or/1–10
12.	exp Hospitalization/
13.	(in-patient or hospitalized or hospitalised or hospitalisation or hospitalization).tw.
14.	exp Outpatient Clinics, Hospital/
15.	in-hospital.tw.
16.	exp Hospital Units/
17.	(Patient Admission or patient readmission or patient readmission or discharge).tw.
18.	or/12–17
19.	11 and 18
20.	randomized controlled trial.pt.
21.	controlled clinical trial.pt.
22.	intervention studies/
23.	experiment$.tw.
24.	pre test or pretest or (posttest or post test)).tw.
25.	random allocation/
26.	or/20–25
27.	18 and 26

Furthermore, we employed citation tracking, which examines included studies and previous reviews and contacted investigators to identify any study missed by the electronic searches.

### Quality assessment and data analysis

For quality of studies (see additional File 1), we adhered to the Effective Organisation of Care Group (EPOC) module[15] and defined three risk classes: Class I (low risk of bias), Class II (moderate risk of bias) and Class III (high risk of bias). Two reviewers independently assessed and abstracted data, on the intervention criteria, study characteristics and methodological quality. Any disagreement was discussed with a third reviewer. Studies with a high risk of bias were excluded from the review after documentation. If a primary study did not provide information about the standard deviation, we used the approximative or direct algebraic connection between the stated confidence intervals, or p-values, and the standard deviation and calculated the inverse transformation to the individual or pooled standard deviation [12]. Prior to the actual statistical pooling of the singular effects, an adjustment of the data regarding costs due to inflation and price adjustment (OECD Health Care Price Index) was carried out [16]. We chose the US Dollar (USD) as the basic currency. The year 2000 was chosen as a representative year for inflation and price adjustments or exchange rates.

We used Review Manager (RevMan) of the Cochrane Collaboration to calculate a pooled effect estimate, called weighted mean difference (WMD) [17]. We used a random effects model since this model estimates the effect with consideration to the variance between studies, rather than ignoring heterogeneity by employing a fixed effect model. The effect sizes were generated using a model fitting inverse variance weights [17].

### Heterogeneity and meta-analysis

Despite the expected clinical heterogeneity (clinical variability of the included pathway interventions) within the review, it is important to assess the comparability of the results from individual studies. A useful statistic for quantifying inconsistency is I^2 ^= [(Q df)/Q] × 100%, where Q is the chi-squared statistic and df is its degrees of freedom [12]. This quantifies the total variance explained by the heterogeneity as a percentage. We considered an overall test-value greater than 60% to serve as evidence of substantial heterogeneity of a magnitude were statistical pooling is not appropriate.

### Sensitivity analysis

In a sensitivity analysis, both fixed effects and random effects models were employed to determine the causes of heterogeneity and test the confidence that can be placed in both estimates. Only robust estimations of the pooled effects with similar results in fixed effects and random effects models are included in the meta-analysis and discussed. Furthermore, sensitivity analyses were performed to test whether the effect size varied by the countries where the study was carried out (adjusting for market forces) and the year of publication, adjusting for temporal trends.

### Subgroup analysis

We decided previously to perform a subgroup analysis of invasive versus non-invasive clinical pathways, whereupon the distinction between invasive or non-invasive interventions refers to the nature of patient management guided by clinical pathways (i.e. clinical pathways for gastrectomy; transurethral resection of the prostate; hip and knee arthroplasty; percutaneous transluminal coronary angioplasty; etc. versus clinical pathways for asthma care; stroke; pneumonia; etc.). According to theories on health economics, invasive procedures can be standardized more easily than treatment strategies in conservative sectors due to the lower treatment variance [18].

### Assessing publication bias

We used a funnel plot analysis to assess publication bias, i.e. the bias caused by a lower likelihood of publication for non-significant studies. The funnel plot is a scatter-plot with the x-axis representing the effects estimated from the primary studies, and the y-axis representing a measure of the sample size in each study (SE; standard error of the mean) [19]. If publication bias is absent, the diagram shows an inverted symmetrical funnel.

However, given that publication bias poses a threat to the validity of this meta-analysis and the graphical method is subjective in nature, we also applied a statistical approach, often called fail-safe N test.

This test provides an estimate of the number of unpublished or in this context called file-draw studies (Nfs) having an average of no effect that is necessary in the analysis for reducing the pooled effect size from significant to non-significant [20]. Using a critical d level (d crit) of -0.20, the estimate of unpublished studies was calculated with

Nfs = (Ntotal (mean d - d crit))/d crit;

where Ntotal is the total number of studies included in the Meta-Analysis and d is the overall pooled effect (WMD).

### Secondary analyses

The distribution of the length of stay is limited downwards in a natural way (because of the minimum value is always 0), whereas upwards the values can scatter significantly. According to logarithmic transformation, values with this characteristic can have an approximately normal distribution [21]. In the additional File 2 we detailed the formulas used for log transformation. Data analyses were performed using SPSS version 15.0 [22].

## Results

### Search strategy and intervention characteristics

**Figure 1:**
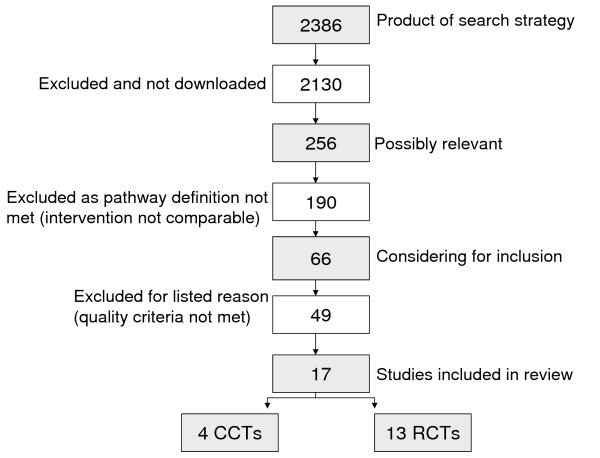
**Identification of relevant studies/trail flow**. [see attached file 1] (PDF Format).

**Figure 2:**
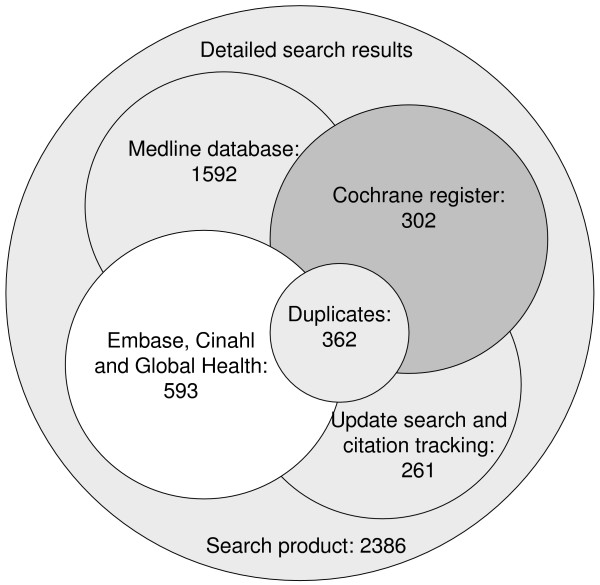
**Electronic search results**. [see attached file 2] (PDF Format).

The specialised search strategy led to the initial selection of 2,386 studies, whereas only 17 matched our methodical requirements (see Figure 1 &2). For the first stage of the study assessment, we scanned all of the 2,386 titles and abstracts for inclusion, the remaining 256 possibly relevant studies were retrieved as full text articles. Based on the full text assessment, we excluded 190 studies out of 256 because they failed to meet our pathway definition. The majority of the excluded studies failed to meet the multidisciplinary pathway criterion, i.e. it was a therapy guideline issued by a medical association or it was a uni-professional nursing care plan. Others did not meet the "algorithm or protocol based" criterion because there was no structure and detailed care plan. For example a poster with issued guidelines was posted in the Emergency Department.

Evidence for meeting the minimal criterion "pathway content is minimally based on one RCT" was reported in 15 studies out of 17, which were included in the review. The study from Falconer et al. and Swanson at al. met the evidence criterion, "not sure" because we failed in contacting the principal investigators [23,24].

### Intervention characteristics

The reported pathway strategies can be described as complex pathway interventions versus a "non-intervention" control group or often poorly, described as "usual or traditional care" group. Most of the experimental interventions were combined with other types of interventions like audit and feedback, educational meetings, and reminders. For 8 out of the 17 (47%) included studies, it was clear that the structured care plan was combined with a "clinical diagnostic or assessment protocol"[23,25-31].

The evidence base for two (12%) pathway interventions was "not sure," whereas the remaining 15 interventions were minimally based on one randomized study or good evidence. The reported purpose of the pathway strategies was appropriate management or cost containment.

The hospital setting was in two studies a multi-center study comprising a range of hospitals included in the investigation [26,30]. From the 15 remaining single center studies, 8 studies (53%) were carried out in a university (teaching) hospital setting [24,27-29,32-35] and 7 studies (46%) in a non-university hospital setting [23,25,31,36-39].

Details of the intervention characteristics are given in Table 3.

**Table 3:** Characteristics of studies included

**Study-ID**	**Study Quality**	**Country**	**Sample Size [N]**	**Mean Age [Years]**	**Diagnosis/Intervention**
**Invasive Care**					

Grines, CL 1998	Class I	USA*	471	56	Primary Angioplasty in Myocardial Infarction
Swanson, CE 1998	Class II	Australia	67	55	Femoral Fractures
Dowsey, MM 1999	Class II	Australia	163	66	Hip and Knee Arthroplasty
Choong, PF 2000	Class II	Australia	111	81	Fractured Neck of Femur
Aizawa, T 2002	Class II	Japan	69	71	Transurethral Resection of the Prostate
Kiyama, T 2003	Class II	Japan	85	63	Gastrectomy
Hirao, M 2005	Class II	Japan	103	61	Gastrectomy

**Non-Invasive Care**					

Falconer, JA 1993	Class II	USA*	121	68	Stroke Rehabilitation
Gomez, MA 1996	Class I	USA*	100	52	Myocardial Ischemia
Roberts, RR 1997	Class II	USA*	165	48	Chest Pain
Johnson, KB 2000	Class II	USA*	110	7	Paediatric Asthma
Kollef, HM 2000	Class II	USA*	489	60	Respiratory Care
Marrie, TJ 2000	Class I	USA*	19***	64	Community-Acquired Pneumonia
Sulch, D 2000	Class II	UK**	152	75	Stroke Rehabilitation
Kim, MH 2002	Class II	USA*	18	48	Artrial Fibrillation
Chen, SH 2004	Class II	Taiwan	42	8	Paediatric Asthma
Usui, K 2004	Class II	Japan	61	48	Community-Acquired Pneumonia

Note: USA* = United States of America; UK** = United Kingdom; 19*** = (19) hospitals at random (1743 Patients)

### Quality assessment

To summarize, we examined the design and study quality of 66 studies, excluding 49 out of the 66 studies because of the high risk of bias (see trail flow, Figure 1). Characteristics (reason for exclusion: see additional File 3) of the 49 excluded studies (references to excluded studies: see additional File 4) are given in detail.

The patient was randomized to the experimental or control group in 12 out of 13 (92%) RCTs. The randomization process was clear in all such studies and justified by the authors. Referring to the individually randomized and single-center studies, the assessment of protection against contamination of the control professionals remained unclear due to poor reporting. None of the investigators reported protection against contamination (communication between experimental and control professionals) and it is possible that control subjects received the intervention. Only the investigation from Marie et al. used a robust cluster randomized design, with 19 hospitals as unit of allocation [30]. To avoid "unit of analysis error," we conducted the meta-analysis at the same level as the allocation (19 cluster-hospitals = 19 patients).

Poor reporting also lead to difficulties in determining the assessment of the power calculations. For instance, sample-size calculation was unclear for over 60% of the included studies; hence the study sample may not have been sufficiently large. Another problem, due to poor reporting was the selection of comparators. The choice of the comparator (i.e. the control and intervention units were located either in the main building or the east building of the participating hospital) was stated and justified by the authors of the 17 primary studies. However, a clear description of what was meant by traditional care or usual care (control group) would have helped in assessing the relevance of the study to other settings.

Primary studies reporting economic data, can be described by a very limited scope of evaluation, focusing on direct hospital LOS and costs effects, rather than on a full economic evaluation [16]. In Table 3 the quality assessment and characteristics of the 17 studies included in the review and meta-analysis are shown in detail.

### Effects on LOS

**Figure 3:**
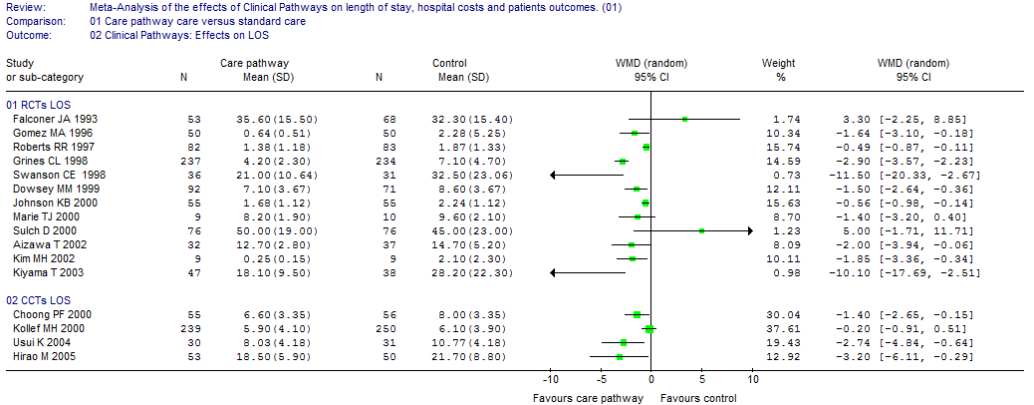
**Effects on LOS**. [see attached file 3] (BMP Format).

Out of the 16 studies (12 randomized and four non-randomized studies representing a study population of 4,028 patients) examining the effect of clinical pathways on the length of stay, 12 showed significant effects [23,24,26-39]. However, heterogeneity between studies reporting on LOS was substantial (I^2 ^= 80%) and may refer to both the statistical inconsistency as well as to the varying clinical pathway interventions that were included. As a result, the estimation of an overall pooled effect is not appropriate and in Figure 3, the differences from the individual studies in LOS are depicted together with the corresponding confidence intervals without totals. The reported LOS in Kiyama 2003 was calculated from the day of surgery to the day of discharge [39]. All other studies included in this analysis considered the total LOS.

### Effects on patient outcomes

**Figure 4:**
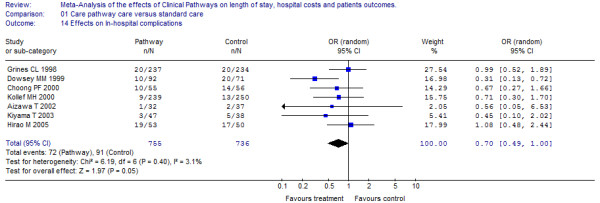
**Effects on in-hospital complications**. [see attached file 4] (BMP Format).

**Figure 5:**
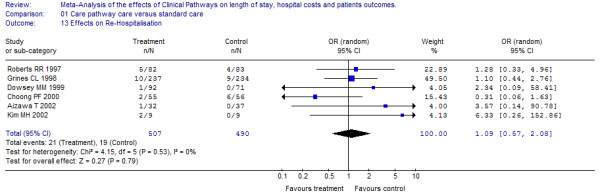
**Effects on re-hospitalisation**. [see attached file 5] (BMP Format).

Out of 17 trials reporting effects on quality outcome measures (see Table 1); six measures were comparable in terms of re-hospitalisation and seven in terms of in-hospital complications [23-35,37-39]. In total, nine primary studies were included in the Meta-analysis (representing a study population of 1,674 patients), examining the effect of clinical pathways on quality patient outcomes. The pooled Odds Ratio (OR) for re-admission was 1.1 (95% CI: 0.57 to 2.08) and for in-hospital complications the overall OR was 0.7 (95% CI: 0.49 to 1.0). Statistical heterogeneity was not present among the studies and there was no evidence of difference in readmission to hospitals or in-hospital complications. The effects of clinical pathways on clinical outcomes in the individual studies are depicted together with the pooled OR (see Figure 4 &5). There was clinical variance in the range of follow-up periods that were used by the investigators measuring re-hospitalization (follow up periods ranged from 27 days to 6 month, see Table 1) as well as the investigators used varying definitions of the term in-hospital complications (included in-hospital complications were cardiac events, infections, thrombosis, re-operation, sepsis and empyema). Obviously, this implies that any time element in the patient outcome data is lost through this approach and it was not possible to compute a series of dichotomous outcomes, i.e. at least one event during the first year of follow up.

### Effects on hospital costs

Six of the included studies (five randomized and one non-randomized), representing a sample of 1328 treated patients, reported on cost effects [26,28,31,33,34,39]. Four out of the five randomized studies found significantly lower hospitalisation costs for pathway groups. The statistical heterogeneity was substantial (I^2 ^= 88%) and compromised the estimation of a pooled effect. Additionally, we also observed a considerable methodological variation which refers to the different methods of cost calculation used by the investigators. Some investigators used a full cost approach (fix and variable costs included), whereas others calculated only direct hospital costs. Table 4 describes the costs differences in detail.

**Table 4:** Cost data, standardized to the year 2000

**Study ID**	**Country**	**Currency**	**Experiment**	**SD**	**Control**	**SD**
Kiyama, T 2003	Japan	US$	$14013	$2634	$18020	$7332
Kim, MH 2002	USA*	US$	$879	$394	$1706	$1512
Kollef, HM 2000	USA*	US$	$922	$1614	$1120	$1430
Grines, CL 1998	USA*	US$	$11430	$6257	$13733	$7249
Roberts, RR 1997	USA*	US$	$1877	$1243	$2574	$999
Gomez, MA 1996	USA*	US$	$1535	$1985	$6768	$17359

Note: USA* = United States of America

### Subgroup analysis: invasive versus non-invasive clinical pathways

**Figure 6:**
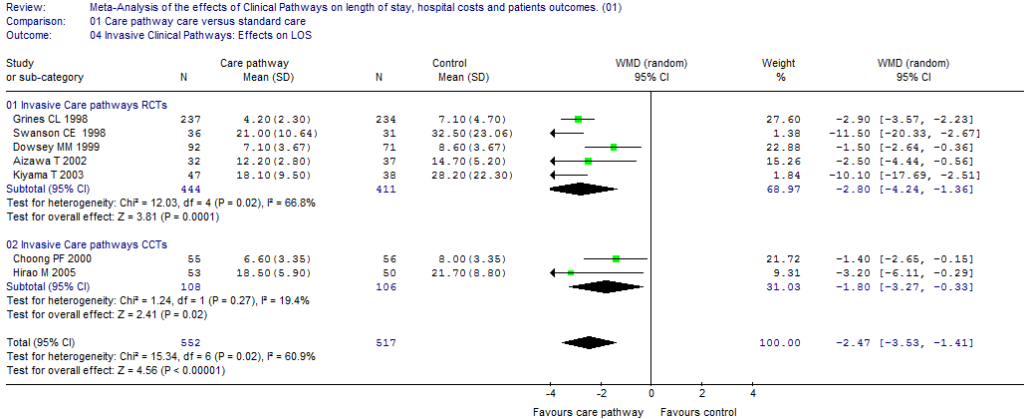
**Effects on LOS invasive pathways**. [see attached file 6] (BMP Format).

Five of the randomized studies, and two further non-randomized studies assessed the LOS effects of surgical or minimally invasive interventions [24,26,27,29,35,37,39]. The pooled effect for all invasive primary studies was -2.5 days (95% CI: -3.53 to -1.41). The differences in LOS in the individual studies are depicted together with the total effect per study type (RCTs versus CCTs, Figure 6). The statistical pooling of the subgroup of surgical pathway interventions is characterized by a considerable overall test-value for statistical inconsistency (I^2 ^= 60.9%) which also reflects the clinical heterogeneity of the surgical pathway interventions included in this comparison.

**Figure 7:**
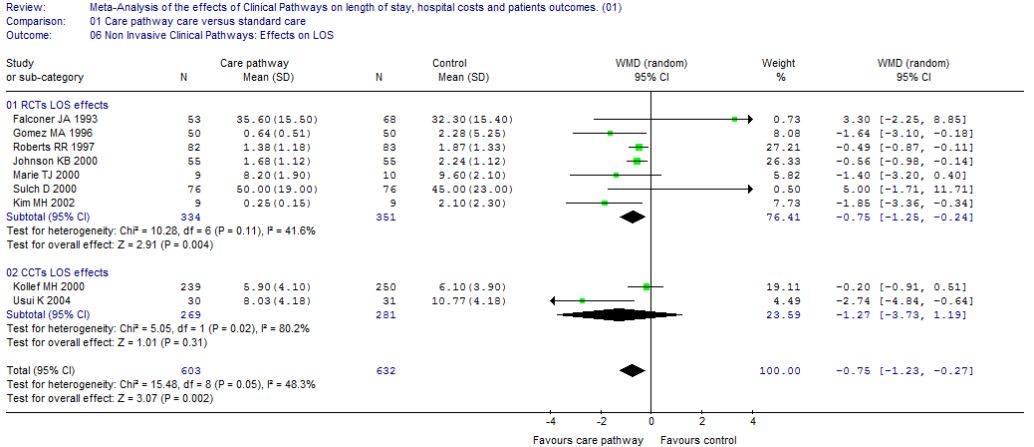
**Effects on LOS non-invasive pathways**. [see attached file 7] (BMP Format).

The subgroup of the conservative pathway indications [23,28,30-34,36,38] had a reduction of LOS of approximately one day (WMD -0.75; 95% CI: -1.23 to -0.27, Figure 7).

### Sensitivity analyses

The LOS effects were robust in terms of the sensitivity analysis concerning the different statistical calculation models (fixed versus random effects model) and the Year of publication, adjusting for temporal trends. However, we observed a trend toward greater reported LOS effects from Japanese studies with a reduction of approximately three days (WMD – 2.7), followed by studies carried out in Australia (WMD – 1.5), Canada (WMD – 1.4) and the USA (WMD – 0.8). Subsequently, we tested the hypotheses, that different market forces (reported effect sizes per country) are possibly confounding the conclusions of these review and meta-analysis. After exclusion (stepwise/iterative and all of the primary Japanese studies) of the subgroup of Japanese studies, the (calculative) overall LOS effect remained robust and statistically significant, but tended to be smaller (WMD – 1.2; subgroup "Japanese studies excluded" versus WMD – 1.5; subgroup "all primary studies" included). This applies also to the subgroup Analysis "Invasive versus non-invasive LOS effects", after exclusion of the subgroup of Japanese studies (WMD -0.6 conservative versus -2.2 invasive).

In addition, the overall odds ratios (OR) for re-admission and in-hospital complications were robust in all terms of the sensitivity analysis, indicating reliable pooled results.

### Publication bias and other sources of systematic error

**Figure 8:**
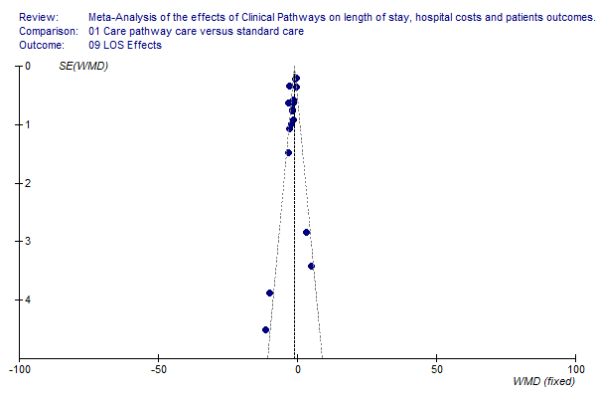
**Funnel plot analyses**. [see attached file 8] (BMP Format).

The funnel plot showed a relatively symmetric distribution (Figure 8), but the point cloud does not have a distinctive funnel form. The deficient funnel form of the funnel plot can also be due to the relatively high heterogeneity with respect to the different pathway indications of the primary studies included in these review (cross-indicational methodology of the primary studies). Furthermore, the number of studies was relatively small. However, given that publication bias may still exist, the statistical fail-safe N objectively helps to quantify. The calculation about the number of file-drawer studies showed that 101 non-significant studies would have to exist to reduce the (calculative) overall effect size of (WMD) – 1.47 to a mean effect size of – 0.2. These results indicate that unpublished research is unlikely to threaten the validity of the original meta-analysis.

### Secondary analyses

**Figure 9:**
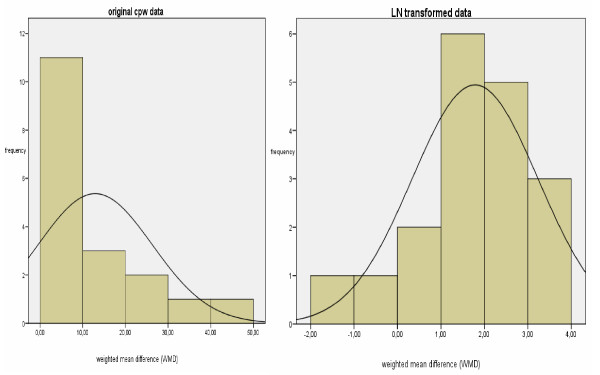
**a & b – Graphic distribution original cpw data and LN transformed**. [see attached file 9 a & b] (PDF Format).

The graphic distribution of the original and the logarithmical (natural logarithm LN) LOS data clearly indicates that there is a significant deviation from the normal distribution (Figure 9a &9b). The heterogeneity between studies was not substantially lowered by the log transformation.

The LOS reduction was estimated as 25 percent (95% CI: -36% to -14%). For example, with the average length of stay of 7 days, the estimated LOS effect was approximately -1.7 days (7 days * -0.25 = -1.75 days LOS reduction). Furthermore, the pooled percentage LOS effects were similar in fixed versus random effects models (WMD -0.25 versus WMD -0.21).

## Discussion

In general, the majority of studies reporting economic data (LOS and hospital costs) showed a positive impact. The results suggest that clinical pathways for invasive procedures generate clearer LOS effects (WMD -0.8 conservative versus -2.5 invasive).

Clinical pathways appeared to be effective in reducing LOS and costs. Furthermore, pathways for invasive procedures showed a stronger LOS reduction by comparing the magnitudes of effect. These results may not be applied for acute rehabilitation for stroke, where reverse effects were reported (see effects on LOS, Figure 3) [23,38]. Both trends were not statistically significant but they were in contrast to the majority of pathway effects reported in the present review. However, the question of comparability may rise as a reflection of the differing pathway components included in this review and also applies to the kind and number of providers included in the primary studies.

We did not publish our review protocol prior to the study. The review protocol for the follow-up study will be published as a Cochrane review to prevent any doubt about the comparison to be data-driven instead of protocol-driven. We determined the scope of this review question on a pilot analysis of existing primary study data resulting in a diverse set of included studies. As it is an explanatory analysis, the pooled results of the meta-analyses may only apply for the majority of included pathway conditions reporting positive effects or trends. Another limitation refers to the poorly described control conditions reported in the primary studies and implied both, the risk of contamination, and the masking of effects. Therefore, we did not pool the primary LOS and costs data from all of the 17 included studies and concentrated on examining the relationship between clinical subgroups (i.e. surgical versus non-invasive pathway conditions).

It should be noted that the development and implementation of clinical pathways consumes a considerable amount of resources. This corresponds to the fact that truly achievable costs savings depend on the number of cases (volume). This has to be included in the costs analysis. The inflation-adjusted costs for implementation (without maintenance and further development) of the pathway indication "Caesarian section" amounted to nearly $20,000 [40]. However, since normally 20 percent of the diagnoses cover 80 percent of the cases [18], a considerable percentage of medical services can be dealt with using a relatively small number of clinical pathways. Therefore, the expenditures will amortize rapidly.

It is very important not to look too far into these results, as there were some limitations. Moreover, it has to be emphasised that evidence determined by meta-analysis is always exploratory in nature and should be considered with caution.

Due to the result of the relatively small number of studies meeting inclusion criteria, this evidence base is not conclusive enough to provide a replicable framework for all pathway strategies. Considering the clinical areas for implementation, clinical pathways seem to be effective especially for invasive care. The likely benefits and costs need to be considered by the local healthcare providers when implementing clinical pathways under different circumstances. This review has shown that there is not one, singular strong evidence base. Accordingly, decision-makers should also consider some limitations in relation to the generalization of these findings. Replicating the results of this review in other settings could be problematic (e.g. ceiling effects such as market forces).

The heterogeneity in design and outcomes of the studies was large and refers to the statistical heterogeneity in addition to the clinical variability of the included studies. This precluded the overall pooling of LOS and cost data, although the order of magnitude of effects indicated that there are considerable implications of using clinical pathways.

It is unavoidable that some studies will have been overlooked, despite our electronic search strategy. Studies meeting our clinical pathway definition (see Table 1 &4) were included, regardless of the fact that the term pathway was mentioned in the study and was done to avoid subjectivity. Also, studies were independently assessed and data extracted by two with any disagreement discussed with a third reviewer.

Finally, should be emphasised that the standard of the primary studies included pose a threat to the validity of the results. While the overall quality of the included studies was moderate, most demonstrated methodological weaknesses such as a small sample size available for analysis.

## Conclusion

With respect to the totality of available evidence, the knowledge about the mechanisms through which pathways work is insufficient. Future research should focus on a better understanding of the key elements of clinical pathways that have impact on economic and patient outcomes. It is also surprising that more studies do not consider any cost effects other than those of treatment. Health-economic research should therefore concentrate on costs of development and implementation of clinical pathways.

This investigation is the first systematic review regarding the effects of clinical pathways on process and patient outcomes. We explicitly decided to expand this review and will also include less restrictive study designs in addition to randomized and quasi-randomized trials, to provide a comprehensive theoretical basis. The character of non-experimental studies makes them even more difficult to critically assess and moreover, due to the lack of MeSH terms the search results cannot be as sensitive as those for purely RCT/CCT-based reviews. Another future direction is a more comprehensive, patient-centered approach, concentrating more on patient-outcomes rather then health-economic study endpoints. The next scheduled update for this review is planned for the End of 2009.

## Competing interests

The authors declare that they have no competing interests.

## Authors' contributions

TR: Made substantial contributions to conception and design, acquisition of data, analysis and interpretation of data. In particular, he independently screened all titles and abstracts in the first stage of study assessment and led the study assessment of all full text papers as well as the data extraction. He has also been involved in drafting the manuscript and revised it critically as corresponding author. JK: Made substantial contributions to conception and design, acquisition and interpretation of data. He has also been involved in drafting the manuscript, revised it critically for important intellectual content and given final approval. RK: Made substantial contributions to quality assessment, analysis and interpretation of data. In particular, he also independently screened all abstracts, assessed the full text papers and double checked the extracted study data. Has also been involved in drafting the manuscript, revised it critically for statistical content and given final approval. HG: Made substantial contributions to conception and design, acquisition of data and revised the manuscript critically. He has also been involved in developing the electronic-search strategy and any disagreement between TR and RK was discussed with him as a third party reviewer. ST: Has been involved in acquisition of data, screening of studies, retrieving potentially relevant studies and has been involved in drafting the manuscript. JMO: Has been involved in screening of studies, retrieving potentially relevant studies, drafting the manuscript and revised it critically for statistical content. EWS: Made substantial contributions to acquisition of data, statistical analysis and interpretation of data. Has also been involved in drafting the manuscript, revised it critically for intellectual content and given final approval.

## Pre-publication history

The pre-publication history for this paper can be accessed here:



## Supplementary Material

Additional file 1 Quality criteria for RCTs and CCTs. The depicted quality criteria represent the standard used for quality assessment Click here for file

Additional file 2 Formulas log transformation. The depicted formula was used for log transformation Click here for file

Additional file 3 Characteristics of excluded studies. The table briefly reflects the characteristics of excluded studies and the reasons for exclusion Click here for file

Additional file 4 References to excluded studies. The table depicts the references to excluded studies Click here for file
