# Supplementary material for: A systematic review and meta-analysis of the effects of clinical pathways on length of stay, hospital costs and patient outcomes
Source: BMC Health Serv Res. 2008 Dec 19;8:265. doi: 10.1186/1472-6963-8-265 (PMC2632661; doi:10.1186/1472-6963-8-265)
Supplement: Additional file 1 — Quality criteria for RCTs and CCTs. The depicted quality criteria represent the standard used for quality assessment [file 1472-6963-8-265-S1.pdf]

Quality criteria for RCTs and CCTs

QUALITY CRITERIA	DONE	NOT CLEAR	NOT DONE
(a) Concealment of allocation	Unit of allocation was institution, team or professional and any random process explicitly described, e.g. use of random number tables, OR unit of allocation was patient or episode of care and some form of centralised randomisation scheme, an on-site computer system or sealed opaque envelopes used	Allocation procedure not described explicitly OR unit of allocation was patient or episode of care and reported use of 'list' or 'table', 'envelopes' or 'sealed envelopes' for allocation	Use of alternation, such as reference to case record numbers, dates of birth, day of the week or any other such approach OR unit of allocation was patient or episode of care and reported use of any allocation process that is entirely transparent before assignment, such as an open list of random numbers or assignments OR allocation was altered by investigators, professionals or patients
(b) Follow-up of professionals (protection against exclusion bias)	Outcome measures for ≥ 80% of professionals randomised (Do not assume 100% follow-up unless stated explicitly)	Not specified	Outcome measures for < 80% of professionals randomised
(c) Follow-up of patients or episodes of care.	Outcome measures for ≥ 80% of patients randomised or patients who entered the trial (Do not assume 100% follow-up unless stated explicitly)	Not specified	Outcome measures for < 80% of patients randomised or patients who entered the trial
(d) Blinded assessment of primary outcome(s) (protection against detection bias)	Stated explicitly that primary outcome variables were assessed blindly OR outcome variables are objective, e.g. length of hospital stay, drug levels assessed by a standardised test	Not specified	Outcomes not assessed blindly
(e) Baseline measurement	Performance or patient outcomes measured prior to the intervention, and no substantial differences present across study groups	Baseline measures not reported, or unclear whether baseline measures are different across study groups	Differences at baseline in main outcome measures likely to undermine the post-intervention differences, e.g. differences between groups before the intervention similar to those found post-intervention
(f) Reliable primary outcome measure(s)	Two or more raters with agreement ≥ 90% or kappa ≥ 0.8 OR outcome assessment is objective, e.g. length of hospital stay, drug levels assessed by a standardised test	Reliability not reported for outcome measures obtained by chart extraction or collected by an Individual	Two or more raters with agreement < 90% or kappa < 0.8.
(g) Protection against contamination	Allocation by community, institution or practice and unlikely that control group received the Intervention	Professionals allocated within a clinic or practice and possible that communication between experimental and control group professionals could have occurred	Likely that control group received the intervention, e.g. cross-over trials or if patients rather than professionals were randomised
Risk of bias:	Class I (Low risk) = all criteria checked as "Done"	Class II (Moderate risk) = all criteria checked as "Done" or "Not clear"	Class III (High risk) = one or more criteria checked as "Not done"

Legend: Except criterion (a) random process, the quality criteria apply for RCTs and CCTs. Source: Bero L, Grilli R, Grimshaw JM, Mowat G, Oxman A, Zwarenstein M: Cochrane Effective Practice and Organisation of Care Review Group (Cochrane Group Module). In: Bero L, Grilli R, Grimshaw JM, Mowat G, Oxman A, Zwarenstein M. Oxford: The Cochrane Library (Issue 3); 2001
