# Supplementary material for: A systematic review and meta-analysis of the effects of clinical pathways on length of stay, hospital costs and patient outcomes
Source: BMC Health Serv Res. 2008 Dec 19;8:265. doi: 10.1186/1472-6963-8-265 (PMC2632661; doi:10.1186/1472-6963-8-265)
Supplement: Additional file 2 — Formulas log transformation. The depicted formula was used for log transformation [file 1472-6963-8-265-S2.pdf]

Formulas log transformation

Formula log transformation:

Log transformed data were used in the secondary analysis. For the logarithmic transformation of the primary data, we used the following formulas:

$\sigma^2 = \ln(s^2/m^2+1)$; $\mu = \ln(m/\text{radical}(s^2/m^2+1))$;

where μ and σ^2 are the mean and variance on the log transformed scale, and m and s^2 the mean and variance on the original scale respectively.
