# Supplementary material for: A systematic review and meta-analysis of the effects of clinical pathways on length of stay, hospital costs and patient outcomes
Source: BMC Health Serv Res. 2008 Dec 19;8:265. doi: 10.1186/1472-6963-8-265 (PMC2632661; doi:10.1186/1472-6963-8-265)
Supplement: Additional file 3 — Characteristics of excluded studies. The table briefly reflects the characteristics of excluded studies and the reasons for exclusion [file 1472-6963-8-265-S3.pdf]

Characteristics of excluded studies

Study	Reason for exclusion
Abe 2001	The investigators compared two cohorts (short and long program). Study design: high risk of bias.
Bailey 1998	The investigators used a pre- post comparison. Study design: high risk of bias.
Board 2000	The investigators used a RCT design to compare "usual hospital care versus home care (hospital in the home). Setting not comparable.
Du Pen 1999	The investigators used a multi-center RCT design to compare "best practice cancer care versus standard practice (usual care). Community setting not comparable.
Fine 2003	The investigators used a seven site cluster RCT design to compare "pneumonia guideline dissemination (via E-Mail)" versus a multifaceted intervention strategy (experimental pneumonia pathway). Design at high risk of bias as a direct result of unit of analyses error.
Fridlin 1996	The investigators used a time series. Study design not met.
Kagan 2002	The investigators used a pre- post comparison. Study design: high risk of bias.
Keetch 1998	The investigators used a (controlled) pre- post comparison. Study design not met.
Kelly 2000	The investigators compared a prospective cohort with a historical matched control. Study design not met.
Kight 1999	The investigators used a (controlled) pre- post comparison. Study design not met.
Kim 2001	The investigators used a pre- post comparison. Study design not met.
Leibman 1998	The investigators used a (controlled) pre- post comparison. Study design not met. .
McKinsey 1999	The investigators used a pre- post comparison. Study design not met.
Melbert 2002	The investigators used a case-control design. Study design not met.
Metersky 2001	The investigators used a pre- post comparison. Study design not met.
Miller 2002	The investigators compared a 5-year cohort with a historical control. Study design not met.

Characteristics of excluded studies (Continued)

Nanly 2005	The investigators used a pre- post comparison. Study design not met.
O'Brien 2000	The investigators used a pre- post comparison with 3 measures in time. Study design not met.
Ogawa 2004	The investigators used a pre- post comparison. Study design not met.
Palmer 2000	The investigators used a cluster RCT design to compare 9 intervention hospitals with 10 control hospitals. The intervention was a pneumonia pathway. Double publication, please see Marrie TJ 2000.
Pearson 2000	The investigators used a pre- post comparison. Study design not met.
Pearson 2001	The investigators used a pre- post comparison. Study design not met.
Perry 2003	The investigators used a pre- post comparison. Study design not met.
Pestian 1998	The investigators used a pre- post comparison. Study design not met.
Peter 2004	The investigators used a pre- post comparison. Study design not met.
Porter 1998	The investigators used a pre- post comparison. Study design not met.
Pritts 1999	The investigators compared an experimental group with a concurrent and a historical control group. Study design not met.
Pronovost 2002	The investigators used a time series. Study design not met.
Ranjan 2003	The investigators used a case-control design. Study design not met.
Riegel 1996	The investigators used a pre- post comparison. Study design not met.
Roberts 2004	The investigators used a (controlled) pre- post comparison. Study design not met.
Roman 2001	The investigators used a pre- post comparison. Study design not met.
Ross 1997	The investigators compared 2 (years) cohorts. Study design: high risk of bias.
Ross 2004	The investigators used a case-control design. Study design not met.
Sanders 2002	The investigators compared 2 patient cohorts from 2 hospitals: high risk of bias.

Characteristics of excluded studies (Continued)

Schriger 1997	The investigators used a time series. Study design not met.
Short 1997	The investigators used a pre- post comparison. Study design not met.
Smith 1999	The investigators used a time series. Study design not met.
Stoller 1998	The investigators used a RCT design. Study quality assessment: Randomization process not reported. Allocation concealment not sure. Study quality: high risk of bias. Quality criteria not met.
Stone 2005	The investigators used a multi-center RCT design. Study quality assessment: High risk of exclusion bias, many patients excluded from the study after randomization. Study quality: high risk of bias.
Summers 1998	The investigators used a time series design. Study design not met.
Thomas 2003	The investigators used a 3year cohort study with a historical control. Study design: high risk of bias.
Turley 1994	The investigators used a case-control design with matched pairs. Study design not met.
Unemura 2002	The investigators evaluated staff satisfaction pre- and post intervention. Study design: measures not objective. High risk of bias.
Walsh 2001	The investigators compared 3 study cohorts in a pre- post comparison. Study design: high risk of bias.
Warner 2002	The investigators used a time series design. Study design not met.
Waters 1999	The investigators used a pre- post comparison with 3 measures reported. Study design: high risk of bias.
Wilson 2002	The investigators used a case-control design with matched pairs. Study design not met.
Yueh 2003	The investigators used a (controlled) pre- post design. Study design: high risk of bias.
