# Supplementary material for: A systematic review and meta-analysis of the effects of clinical pathways on length of stay, hospital costs and patient outcomes
Source: BMC Health Serv Res. 2008 Dec 19;8:265. doi: 10.1186/1472-6963-8-265 (PMC2632661; doi:10.1186/1472-6963-8-265)
Supplement: Additional file 4 — References to excluded studies. The table depicts the references to excluded studies [file 1472-6963-8-265-S4.pdf]

References to excluded studies

Abe 2001

Abe T, Tsuchida N, Ishibashi H, Yamamoto S. [Comparison between the short program and the long program of post-operative rehabilitation of hip fracture for making the critical path]. [Japanese]. Nippon Ronen Igakkai Zasshi - Japanese Journal of Geriatrics.38(4):514-8 2001.

Bailey 1998

Bailey R, Weingarten S, Lewis M, Mohsenifar Z. Impact of clinical pathways and practice guidelines on the management of acute exacerbations of bronchial asthma. Chest.113(1):28-33 1998.

Board 2000

Board N, Brennan N, Caplan GA. A randomised controlled trial of the costs of hospital as compared with hospital in the home for acute medical patients.[see comment]. Australian & New Zealand Journal of Public Health.24(3):305-11 2000.

Du Pen 1999

DuPen SL, DuPen AR, Polissar N, Hansberry J, Kraybill BM, Stillman M, Panke J, Everly R, Syrjala K. Implementing guidelines for cancer pain management: results of a randomized controlled clinical trial. J.Clin.Oncol. 1999;17(1):361.

Fine 2003

Fine MJ, Stone RA, Lave JR, Hough LJ, Obrosky DS, Mor MK, Kapoor WN. Implementation of an evidence-based guideline to reduce duration of intravenous antibiotic therapy and length of stay for patients hospitalized with community-acquired pneumonia: a randomized controlled trial. Am.J.Med. 2003;115(5):343.

Fridlin 1996

Fridlin C. Using severity-adjusted data to impact clinical pathways. Healthcare Information Management 1996;10(1):23-30.

Kagan 2002

Kagan SH, Chalian AA, Goldberg AN, Rontal ML, Weinstein GS, Prior B, Wolf PF, Weber RS. Impact of age on clinical care pathway length of stay after complex head and neck resection. Head & Neck.24(6):545-8; discussion 545 2002.

Keetch 1998

Keetch DW, Buback D. A clinical-care pathway for decreasing hospital stay after radical prostatectomy. British Journal of Urology 1998;81(3):398-402.

Kelly 2000

Kelly RE, Jr., Wenger A, Horton C, Jr., Nuss D, Croitoru DP, Pestian JP. The effects of a pediatric unilateral inguinal hernia clinical pathway on quality and cost. Journal of Pediatric Surgery.35(7):1045-8 2000.

Kight 1999

Kight L. Chest pain rule-out MI clinical pathway saves $183,000. Hospital case management : the monthly update on hospital-based care planning and critical paths 1999;7(12):207-210.

Kim 2001

Kim MH, Deeb GM, Morady F, Bruckman D, Hallock LR, Smith KA, Karavite DJ, Bolling SF, Pagani FD, Wahr JA, Sonnad SS, Kazanjian PE, Watts C, Williams M, Eagle KA. Effect of postoperative atrial fibrillation on length of stay after cardiac surgery (The Postoperative Atrial Fibrillation in Cardiac Surgery study [PACS(2)]. American Journal of Cardiology.87(7):881-5 2001.

Leibman 1998

Leibman BD, Dillioglugil O, Abbas F, Tanli S, Kattan MW, Scardino PT. Impact of a clinical pathway for radical retropubic prostatectomy. Urology 1998;52(1):94-99.

McKinsey 1999

McKinsey KT, Boren DM, Fidellow JA. Navigate a clinical pathway for uncomplicated MI patients. Nursing management 1999;30(10):33-35.

Melbert 2002

Melbert RB, Kimmins MH, Isler JT, Billingham RP, Lawton D, Salvadalena G, Cortezzo M, Rowbotham R. Use of a critical pathway for colon resections. Journal of Gastrointestinal Surgery.6(5):745-52 2002:Oct.

Metersky 2001

Metersky ML, Fine JM, Tu GS, Mathur D, Weingarten S, Petrillo MK, Meehan TP. Lack of effect of a pneumonia clinical pathway on hospital-based pneumococcal vaccination rates. American Journal of Medicine 2001;110(2):141-143.

Miller 2002 Miller PR, Fabian TC, Croce MA, Magnotti LJ, Pritchard FE, Minard G, Stewart RM. Improving outcomes following penetrating colon wounds: Application of a clinical pathway. Annals of Surgery 2002;235(6):775-781.

Nanly 2005

Nanly H, Chen BTH, Lee LL, Chung MH, Lin PC. A comparison of the quality of nursing care between pre- and postimplementing of a clinical pathway. Journal of Clinical Nursing 2005;14(10):1260-1261.

O'Brien 2000

O'Brien SV, Hardy KJ. Impact of a care pathway-driven diabetes education programme. Journal of Diabetes Nursing 2000;4(5):147-9.

Ogawa 2004

Ogawa T, Terada A, Yamada Y, Ijichi K, Hasegawa Y, Fujimoto Y. The meaning clinical pathway of the operation for thyroid tumor and parotid tumor. Practica Oto-Rhino-Laryngologica 2004;97(6):555-561.

Palmer 2000

Palmer CS, Zhan C, Elixhauser A, Halpern MT, Rance L, Feagan BG, Marrie TJ. Pharmaceutical economics & health policy: economic assessment of the community-acquired pneumonia intervention trial employing levofloxacin (Structured abstract). Clinical.Therapeutics 2000;22:250.

Pearson 2000

Pearson S, Moraw I, Maddern GJ. Clinical pathway management of total knee arthroplasty: a retrospective comparative study. Australian & New Zealand Journal of Surgery.70(5):351-4 2000.

Pearson 2001

Pearson SD, Kleefield SF, Soukop JR, Cook EF, Lee TH. Critical pathways intervention to reduce length of hospital stay. [see comments]. American Journal of Medicine 2001;110(3):175-180.

Perry 2003

Perry L, McLaren S. Nutritional support in acute stroke: the impact of evidence-based guidelines. Clinical Nutrition.22(3):283-93 2003.

Pestian 1998

Pestian JP, Derkay CS, Ritter C. Outpatient tonsillectomy and adenoidectomy clinical pathways: an evaluative study. American Journal of Otolaryngology 1998;19(1):45-49.

Peter 2004 Peter S, Fazakerley M. Clinical effectiveness of an integrated care pathway for infants with bronchiolitis. Paediatric nursing 2004;16(1):30-35.

Porter 1998

Porter HB. Health resource utilization and quality of life outcomes of low-risk coronary artery bypass graft patients: a comparison study. Canadian Journal of Cardiovascular Nursing 1998;9(1):10-15.

Pritts 1999

Pritts TA, Nussbaum MS, Flesch LV, Fegelman EJ, Parikh AA, Fischer JE. Implementation of a clinical pathway decreases length of stay and cost for bowel resection. Annals of Surgery 1999;230(5):728-733.

Pronovost 2002

Pronovost PJ, Jenckes M, To M, Dorman T, Lipsett PA, Berenholtz S, Bass EB. Reducing failed extubations in the intensive care unit. Joint Commission Journal on Quality Improvement.28(11):595-604 2002.

Ranjan 2003

Ranjan A, Tarigopula L, Srivastava RK, Obasanjo OO, Obah E. Effectiveness of the clinical pathway in the management of congestive heart failure. Southern medical journal 2003;96(7):661-663.

Riegel 1996

Riegel B, Gates DM, Gocka I, Medina L, Odell C, Rich M, Finken JS. Effectiveness of a program of early hospital discharge of cardiac surgery patients.[see comment][erratum appears in J Cardiovasc Nurs 1997 Apr;11(3):1]. Journal of Cardiovascular Nursing.11(1):63-75 1996.

Roberts 2004

Roberts HC, Pickering RM, Onslow E, Clancy M, Powell J, Roberts A, Hughes K, Coulson D, Bray J. The effectiveness of implementing a care pathway for femoral neck fracture in older people: a prospective controlled before and after study. Age and Ageing 2004;33(2):178-84.

Roman 2001

Roman SH, Chassin MR. Windows of opportunity to improve diabetes care when patients with diabetes are hospitalized for other conditions. Diabetes Care.24(8):1371-6 2001.

Ross 1997

Ross G, Johnson D, Kobernick M. Evaluation of a critical pathway for stroke.[see comment]. Journal of the American Osteopathic Association.97(5):269-272, 275-6 1997.

Ross 2004

Ross MA, Davis B, Dresselhouse A. The Role of an Emergency Department Observation Unit in a Clinical Pathway for Atrial Fibrillation. Critical Pathways in Cardiology 2004;3(1):8-12.

Sanders 2002

Sanders DS, Carter MJ, D'Silva J, James G, Bolton RP, Willemse PJ, Bardhan KD. Percutaneous endoscopic gastrostomy: a prospective audit of the impact of guidelines in two district general hospitals in the United Kingdom. American Journal of Gastroenterology.97(9):2239-45 2002.

Schriger 1997

Schriger DL, Baraff LJ, Rogers WH, Cretin S. Implementation of clinical guidelines using a computer charting system. Effect on the initial care of health care workers exposed to body fluids.[see comment]. JAMA.278(19):1585-90 1997.

Short 1997

Short MS. Charting by exception on a clinical pathway. Nursing management 1997;28(8):45-46.

Smith 1999

Smith DM, Gow P. Towards excellence in quality patient care: A clinical pathway for myocardial infarction. Journal of Quality in Clinical Practice 1999;19(2):103-105.

Stoller 1998

Stoller JK, Mascha EJ, Kester L, Haney D. Randomized controlled trial of physician-directed versus respiratory therapy consult service-directed respiratory care to adult non-ICU inpatients. American Journal of Respiratory & Critical Care Medicine 1998;158(4):1068-1075.

Stone 2005

Stone RA, Mor MK, Lave JR, Hough LJ, Fine MJ. Implementation of an inpatient management and discharge strategy for patients with community-acquired pneumonia. American Journal of Managed Care.11(8):491-9 2005.

Summers 1998

Summers D, Soper PA. Implementation and evaluation of stroke clinical pathways and the impact on cost of stroke care. Journal of Cardiovascular Nursing 1998;13(1):69-87.

Thomas 2003

Thomas K. Clinical pathway for hip and knee arthroplasty. Physiotherapy 2003;89(10):603-609.

Turley 1994

Turley K, Tyndall M, Roge C, Cooper M, Turley K, Applebaum M, Tarnoff H. Critical pathway methodology: effectiveness in congenital heart surgery. Annals of Thoracic Surgery.58(1):57-63; discussion 63-5 1994.

Unemura 2002

Unemura Y, Toriumi H, Nogi H, Saeki T, Shioya H, Misawa T, Yamazaki Y, Fujisaki J, Torii A. Introduction of a unique clinical pathway for colorectal polypectomy and analysis of its value. Tokyo Jikeikai Medical Journal 2002;117(6):419-426.

Walsh 2001

Walsh MD, Barry M, Scott TE, Lamorte WW, Menzoian JO. The role of a nurse case manager in implementing a critical pathway for infrainguinal bypass surgery. Joint Commission Journal on Quality Improvement.27(4):230-8 2001.

Warner 2002

Warner BW, Rich KA, Atherton H, Andersen CL, Kotagal UR. The sustained impact of an evidenced-based clinical pathway for acute appendicitis. Seminars in Pediatric Surgery.11(1):29-35 2002.

Waters 1999

Waters JB, Wolff RS, Blansfield J, Lamorte WW, Millham FH, Hirsch EF. Development and implementation of clinical pathways for the management of four trauma diagnoses. Journal for Healthcare Quality.21(3):4-11; quiz 11 1999:Jun.

Wilson 2002

Wilson SD, Dahl BB, Wells RD. An evidence-based clinical pathway for bronchiolitis safely reduces antibiotic overuse. American Journal of Medical Quality.17(5):195-9 2002:Oct.

Yueh 2003

Yueh B, Weaver EM, Bradley EH, Krumholz HM, Heagerty P, Conley A, Sasaki CT. A critical evaluation of critical pathways in head and neck cancer. Archives of Otolaryngology -- Head & Neck Surgery.129(1):89-95 2003.
